# Association between cardiovascular health and human papillomavirus infection: analysis from NHANES 2005–2016

**DOI:** 10.3389/fpubh.2024.1501409

**Published:** 2024-11-22

**Authors:** Meng Li, Meiying Song

**Affiliations:** Department of Gynecology, Fuxing Hospital, Capital Medical University, Beijing, China

**Keywords:** HPV infection, HR-HPV infection, Life’s Essential 8, cardiovascular health, NHANES

## Abstract

**Background:**

Given the strong association between cardiovascular disease and human papillomavirus (HPV) infection, this study aimed to assess the correlation between HPV infection and cardiovascular health (CVH) as represented by the Life’s Essential 8 (LE8) score.

**Methods:**

This study employed analysis of data obtained from the National Health and Nutrition Examination Survey covering the period from 2005 to 2016. To examine the correlation between the CVH score and both HPV and high-risk HPV (HR-HPV) infections, this research utilized a combination of multivariable regression analysis, smooth curve fitting, and subgroup analysis, following adjustment for pertinent covariates.

**Results:**

This study included a total of 8,264 women, with an average age of 39.53 ± 11.24 years. The HPV prevalence was 43.43% overall, while the HR-HPV prevalence was 17.36%. In the fully adjusted model, an augmentation of 10 points in the CVH score correlated with an 8% reduction in the HPV infection rate [0.92 (0.88–0.96)], and a similar 8% decrease in the HR-HPV infection rate [0.92 (0.87–0.97)].

**Conclusion:**

Our findings indicate that elevated CVH, as denoted by higher LE8 scores, correlates with a decreased of HPV infection rate among U.S. females. The LE8 score shows potential as a shared predictive biomarker for both CVH and HPV infection.

## 1 Introduction

Human papillomavirus (HPV) belongs to the Papovaviridae family, characterized by its double-stranded, circular DNA structure. With over 200 identified types to date (1), is one of the most common pathogens contributing to sexually transmitted infections globally. Transmitted through contact with skin or mucous membranes, HPV manifests a spectrum of clinical diseases in both genders (2, 3). While many HPV-induced conditions are benign, such as various skin warts, persistent infection can precipitate malignancies, notably cervical cancer (4). Moreover, among HPV-infected women, the risk of malignancies affecting extra-cervical organs (e.g., anus, vagina, vulva, oropharynx) is significantly elevated (5). Among women, cervical cancer is the fourth most frequently occurring cancer, trailing breast, colorectal, and lung cancers. Its global prevalence exhibits substantial variability across nations, with incidence rates spanning from under 2–75 cases per 100,000 women (6). During the year 2018, there were 570,000 fresh instances of cervical cancer documented globally, and 311,000 fatalities attributed to the illness (6). Cervical cancer presents a significant public health concern around the world, so it is necessary to investigate cervical cancer-related or HPV infection-related indicators.

Recent research indicates a potential association between cardiovascular diseases (CVD) and HPV. Women infected with HPV exhibit a higher odds ratio (OR) for CVD compared to their non-infected counterparts, with high-risk HPV (HR-HPV) infection further elevating this risk (7, 8). Cervical cancer patients undergoing radiotherapy face higher risks of ischemic stroke and myocardial infarction compared to patients receiving radiotherapy for other diseases (9). Even among patients after radiotherapy for head and neck malignancies, compared with HPV-negative patients, HPV-positive patients had a more than fourfold increased risk of cerebrovascular events (10). On the contrary, for some conventional CVD risk factors, such as age, exercise, smoking, and diet, also exhibit strong correlations with HPV infection (11). These findings emphasize the significance of risk management for patients with HPV infection.

The concept of cardiovascular health (CVH) was introduced by the American Heart Association (AHA) in the year 2010 to assess CVD risk. Initially, CVH was evaluated using the Life’s Simple 7 (LS7) score, comprising seven fundamental health behaviors and factors (12). With advancements in CVH research, scholars’ understanding evolved over time. Consequently, the AHA enhanced LS7 into Life’s Essential 8 (LE8), adding sleep health and improving its evaluation calculations in 2022 (13, 14). LE8 demonstrates notable efficacy in predicting outcomes related to CVD and other major chronic conditions, including life expectancy (14).

Considering the association between HPV infection and CVD itself as well as its risk factors, promoting CVH could potentially help prevent and manage HPV infection. To our knowledge, the correlation between HPV infection and CVH quantized by LE8 score has not been evaluated in research before. Therefore, we investigate the potential correlation between LE8 scores and both HPV infection and HR-HPV infection statuses in this study. Our findings aim to offer novel insights into the management of women’s health.

## 2 Methods

### 2.1 Survey description and participants

The National Center for Health Statistics (NCHS), oversees a large national program called the National Health and Nutrition Examination Survey (NHANES) database (15). Employing a sophisticated multi-stage stratified sampling approach, the survey aims to provide a detailed characterization of the broader U.S. population. The database offers a comprehensive repository comprising sociodemographic profiles, interview transcripts, and health screening results. Leveraging this resource, the NCHS can employ diverse methods to assess the health and nutritional condition of the population in the United States. Moreover, it facilitates the calculation and examination of disease prevalence, along with associated risk factors or indicators. The ethical guidelines were followed during the participant selection process, and the NCHS Research Ethics Review Committee approved the research protocol. Every participant furnished written consent after being fully informed. All research conducted adhered to the principles outlined in the Declaration of Helsinki.

**Figure 1:**
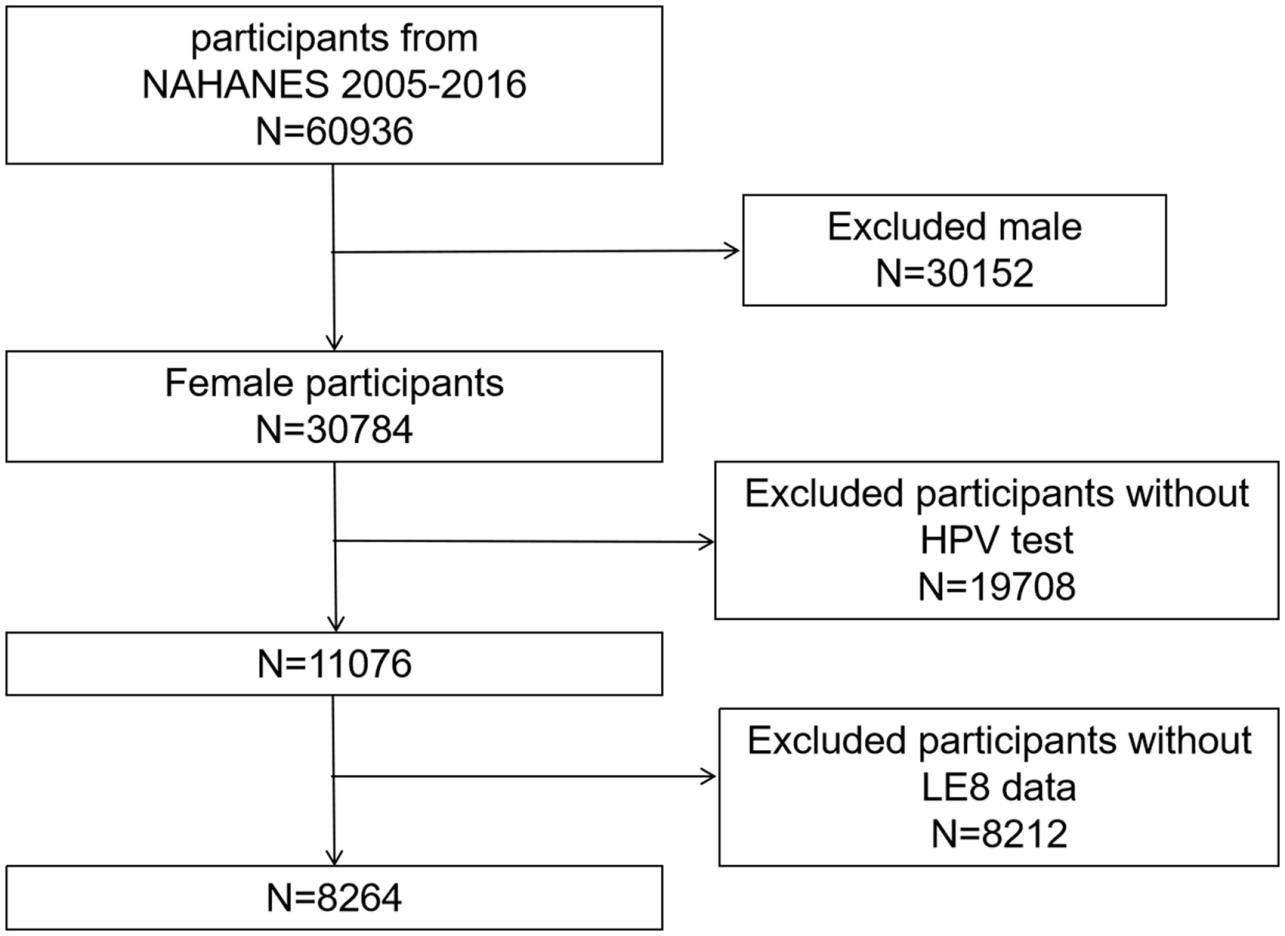
Flowchart.

The data utilized in our study were sourced from NHANES spanning the years 2005 to 2016, encompassing both HPV testing results and all components of the LE8 assessment. The total cohort comprised 60,936 research participants. From this, we excluded 30,152 male participants, 19,708 individuals lacking HPV test results, and 8,212 participants with incomplete LE8 score data. Ultimately, our analysis included 8,264 participants (Figure 1).

### 2.2 Study variables

In this study, the independent variable was the LE8 score, wherein higher scores indicated better CVH. The LE8 score encompassed four health behaviors (diet, physical activity, nicotine exposure, and sleeping health) and four health factors [body mass index (BMI), blood lipids, blood glucose, and blood pressure (BP)]. Dietary indicators were assessed based on participants’ two 24-h dietary assessments, utilizing the Healthy Eating Index (HEI) 2015 (refer to Supplementary Table S1) (16). To gather data on physical activity, nicotine exposure, sleep patterns, medication and diabetes history, self-report questionnaires were employed. Physical examinations were conducted to measure BP, weight, and height. The calculation of body mass index (BMI) involved dividing weight in kilograms by the square of height in meters. For the determination of serum cholesterol, plasma glucose, and hemoglobin A1c levels, blood samples were collected. Each of the eight components of CVH is scored on a scale ranging from 0 to 100 points. To calculate the total LE8 score, the scores of the eight components are summed and then divided by eight, yielding a maximum score of 100 (17). Based on the standards set by the AHA, a LE8 score of 80–100 suggests a high level of CVH, while 50–79 indicates a moderate level, and 0–49 denotes a low level (17). Instructions detailing the computation of LE8 scores using all variables found in NHANES data are provided in Supplementary Table S2 (17, 18).

The dependent variables were the status of HPV infection and HR-HPV infection in this study, assessed through genotyping of DNA obtained from vaginal swabs. The HPV assay employed the Roche Linear Array HPV Genotyping test, while the HR-HPV assay utilized either Hybrid Capture 2 technology or the Cobas test.

Additionally, we investigated potential covariates that might impact the correlation between HPV infection and CVH, comprising age, race, BMI, marital status, education level, family income-to-poverty ratio (PIR), smoking history, age at first sexual intercourse, health insurance coverage, and history of oral contraceptive (OC) use. Data regarding these variables were extracted from the NHANES database, encompassing demographic information, examination records, and self-report questionnaire section.

### 2.3 Statistical analysis

The measurement and demographic characteristics of the study population underwent descriptive analysis. These indicators were stratified into three groups based on varying levels of CVH. Continuous variables were presented as mean ± standard (Mean ± SD), and between-group comparisons were conducted via the *t*-test. Categorical variables were expressed as frequency [n (%)], and compared across groups using the chi-square test. Logistic regression models were used to examine the relationship between LE8 scores and HPV infection rates. The results are presented as odds ratios (OR) with 95% confidence intervals (CI). A univariate logistic regression analysis was performed initially for the crude model. Models 2 and 3 involved multifactorial logistic regression analyses. Specifically, Model 1 remained unadjusted, while Model 2 adjusted for age and race. Moreover, model 3 adjusted for all covariates included in this study. Smoothed curve fitting and threshold effect analysis were employed to further explore the relationship between LE8 scores and HPV infection. Subgroup analyses were conducted based on age, race, education level, marital status, BMI, health insurance status, or smoking history. At last, logistic regression models were employed to investigate the relationship between BP and HPV infection. Model 1 was unadjusted, Model 2 was adjusted for age and race, and Model 3 was adjusted for age, race, BMI, and smoking history. Data were analyzed using Empower software (v.2.0) and the R statistical package (v.3.4.3). Statistical significance was defined as a two-sided *P*-value <0.05.

## 3 Results

### 3.1 Baseline characteristics

Totally, 8,264 women aged 20–59 years were included in this study with an average age 39.53 ± 11.24 years. The overall HPV prevalence was 43.43%, while HR-HPV prevalence was 17.36%. The different CVH subgroups were significantly different with regard to age, race, marital status, education level, PIR, BMI, smoking history, age of first sexual intercourse, and health insurance status (all *P* < 0.05). Nevertheless, CVH subgroups did not exhibit any statistically significant difference in terms of OC using history (*P* > 0.05). As CVH levels rose, there was a corresponding decline in the HPV prevalence, a trend also observed in HR-HPV infections (all *P* < 0.05) (Table 1).

**Table 1:** Baseline characteristics of the study population according to CVH levels.

Characteristics	CVH level			*P*-value
	Low level (LE8: 0–49) *N* = 1,294	Moderate level (LE8: 50–79) *N* = 5,134	High level (LE8: 80–100) *N* = 1836	
Age, years	45.10 ± 9.92	39.44 ± 11.20	35.85 ± 10.64	<0.001
Race, n (%)				<0.001
Mexican American	188 (14.53%)	958 (18.66%)	258 (14.05%)	
Other Hispanic	97 (7.50%)	540 (10.52%)	190 (10.35%)	
Non-Hispanic White	565 (43.66%)	1988 (38.72%)	872 (47.49%)	
Non-Hispanic Black	383 (29.60%)	1,168 (22.75%)	211 (11.49%)	
Other Races	61 (4.71%)	480 (9.35%)	305 (16.61%)	
Marital status, n (%)				<0.001
Married or living with partner	661 (51.08%)	3,044 (59.29%)	1,148 (62.53%)	
Widowed, divorced, or separated	387 (29.91%)	958 (18.66%)	193 (10.51%)	
Never married	246 (19.01%)	1,132 (22.05%)	495 (26.96%)	
Education level, n (%)				<0.001
Less than high school	414 (31.99%)	1,050 (20.45%)	151 (8.22%)	
High school or GED	341 (26.35%)	1,107 (21.56%)	211 (11.49%)	
More than high school	539 (41.65%)	2,977 (57.99%)	1,474 (80.28%)	
PIR	1.79 ± 1.42	2.43 ± 1.62	3.17 ± 1.69	<0.001
BMI, kg/m^2^	36.02 ± 8.29	29.91 ± 7.31	24.08 ± 4.15	<0.001
Age of first sexual intercourse, years	16.89 ± 3.75	17.41 ± 3.44	18.29 ± 3.58	<0.001
Health insurance status, n (%)				<0.001
Has	917 (70.87%)	3,737 (72.79%)	1,499 (81.64%)	
Non	377 (29.13%)	1,397 (27.21%)	337 (18.36%)	
Oral contraceptive taking history, n (%)				0.133
Yes	933 (72.10%)	3,555 (69.24%)	1,285 (69.99%)	
No	361 (27.90%)	1,579 (30.76%)	551 (30.01%)	
Smoked at least 100 cigarettes, n (%)				<0.001
Yes	901 (69.63%)	1848 (36.00%)	267 (14.54%)	
No	393 (30.37%)	3,286 (64.00%)	1,569 (85.46%)	
HPV infection, n (%)				<0.001
Yes	679 (52.47%)	2,257 (43.96%)	653 (35.57%)	
No	615 (47.53%)	2,877 (56.04%)	1,183 (64.43%)	
HR-HPV infection, n (%)				0.002
Yes	269 (20.79%)	860 (16.75%)	306 (16.67%)	
No	1,025 (79.21%)	4,274 (83.25%)	1,530 (83.33%)	

CVH, cardiovascular health; GED, General educational development; PIR, Ratio of family income to poverty; BMI, Body mass index; HPV, Human papillomavirus; HR-HPV, High risk human papillomavirus. Continuous variables were indicated by mean ± SD, assessed via t-tests. Categorical variables were indicated by frequencies [n (%)] and assessed via chi-square tests.

### 3.2 Relationship between CVH and HPV infection

**Figure 2:**
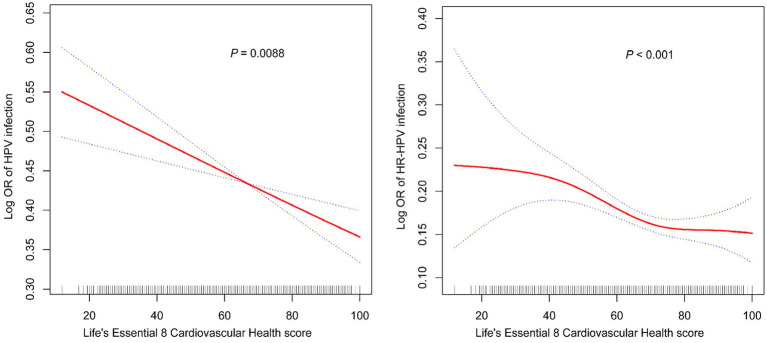
Smooth curve fitting for CVH and HPV infection (A) and HR-HPV (B) infection.

The results of multivariate logistic regression analysis indicated a significant negative correlation between LE8 score and both HPV and HR-HPV infection. In the fully adjusted model, 10 points increase in LE8 score associated with an 8% lower in the odds of HPV infection (OR = 0.92, 95% CI 0.88–0.96); individuals with high levels of CVH exhibited 33% lower HPV infection rates in contrast to individuals exhibiting low levels (OR = 0.67, 95% CI 0.55–0.82). Similarly, 10 points increase in LE8 score was linked to an 8% lower in the odds of HR-HPV infection (OR = 0.92, 95% CI 0.87–0.97) (Table 2). This study confirmed the negative correlation between CVH and HPV infection using a smooth curve fitting approach. This correlation was consistently observed for both total HPV infection (Figure 2A) and HR-HPV infection (Figure 2B).

**Table 2:** The association between CVH and HPV infection.

	Model 1 OR (95% CI) *P*-value	Model 2 OR (95% CI) *P*-value	Model 3 OR (95% CI) *P*-value
HPV infection status			
LE8 scores (per 10 scores)	0.87 (0.84, 0.89) < 0.0001	0.85 (0.83, 0.88) < 0.0001	0.92 (0.88, 0.96) < 0.0001
Low CVH level	Reference	Reference	Reference
Moderate CVH level	0.71 (0.63, 0.80) < 0.0001	0.66 (0.58, 0.75) < 0.0001	0.80 (0.70, 0.93) 0.0032
High CVH level	0.50 (0.43, 0.58) < 0.0001	0.47 (0.40, 0.55) < 0.0001	0.67 (0.55, 0.82) < 0.0001
*P* for trend	< 0.0001	< 0.0001	< 0.0001
HR-HPV infection status			
LE8 scores (per 10 scores)	0.94 (0.90, 0.97) 0.0003	0.89 (0.86, 0.93) <0.0001	0.92 (0.87, 0.97) 0.0017
Low CVH level	Reference	Reference	Reference
Moderate CVH level	0.77 (0.66, 0.89) 0.0007	0.66 (0.56, 0.77) <0.0001	0.74 (0.62, 0.89) 0.0012
High level	0.76 (0.64, 0.91) 0.0034	0.63 (0.52, 0.76) <0.0001	0.77 (0.60, 0.98) 0.0322
*P* for trend	0.0067	< 0.0001	0.0557

Model 1 took no adjustments; Model 2 adjusted for age and race; Model 3 adjusted for all covariates specified. CI, confidence interval; OR, odds ratio.

### 3.3 Relationship between BP and HPV infection

The logistic regression analysis revealed no correlation between BP and HPV infection in both the partially adjusted and fully adjusted models (*P* > 0.05). Only in the unadjusted model, a negative correlation was observed between BP and HR-HPV infection (Supplementary Table S3).

### 3.4 Subgroup analysis

To delve into the potential impact of diverse factors on the correlation between CVH and HPV infection, subgroup analyses were carried out. Upon inclusion of all covariates, subgroup analyses revealed that the relationship between CVH and HPV infection was influenced by marital status and smoking history (*P* for interaction <0.05). Married or cohabiting participants, and those with smoking history, exhibit a stronger negative relationship between CVH and HPV infection (separately OR = 0.87, 95% CI 0.83–0.93; OR = 0.87, 95% CI 0.81–0.93). Furthermore, concerning HR-HPV infection, the correlation with CVH was influenced by marital status and education level (*P* for interaction <0.05). Married or cohabiting participants, as well as those with a high school or General Educational Development (GED) education, displayed a stronger negative relationship between CVH and HR-HPV infection (separately OR = 0.85, 95% CI 0.79–0.92; OR = 0.83, 95% CI 0.74–0.93) (Table 3).

**Table 3:** Subgroups analyses for the association between CVH and HPV infection.

Subgroup	HPV infection status		HR-HPV infection status	
	OR (95% CI)	*P* for interaction	OR (95% CI)	*P* for interaction
Age, years		0.2224		0.9977
Age < 25	1.01 (0.87, 1.16)		0.93 (0.81, 1.08)	
25 ≤ Age < 50	0.91 (0.87, 0.96)		0.94 (0.88, 1.00)	
Age ≥ 50	0.98 (0.90, 1.06)		0.94 (0.84, 1.05)	
Race		0.8261		0.4006
Mexican American	0.92 (0.83, 1.03)		0.92 (0.81, 1.06)	
Other Hispanic	0.96 (0.84, 1.09)		0.98 (0.83, 1.17)	
Non-Hispanic White	0.91 (0.85, 0.97)		0.88 (0.81, 0.96)	
Non-Hispanic Black	0.90 (0.82, 0.98)		0.91 (0.82, 1.01)	
Other Races	0.99 (0.85, 1.15)		1.08 (0.89, 1.32)	
Marital status		0.0309		0.0164
Married or living with partner	0.87 (0.83, 0.93)		0.85 (0.79, 0.92)	
Widowed, divorced, or separated	0.95 (0.87, 1.04)		1.03 (0.93, 1.15)	
Never married	1.00 (0.91, 1.09)		0.94 (0.85, 1.04)	
Education level		0.1559		0.0180
Less than high school	0.97 (0.88, 1.07)		0.85 (0.75, 0.97)	
High school or GED	0.85 (0.78, 0.94)		0.83 (0.74, 0.93)	
More than high school	0.93 (0.88, 0.99)		0.98 (0.92, 1.05)	
BMI, kg/m^2^		0.0539		0.5765
BMI < 25	0.97 (0.90, 1.05)		0.95 (0.86, 1.05)	
25 ≤ BMI < 30	0.85 (0.78, 0.92)		0.95 (0.86, 1.06)	
BMI ≥ 30	0.91 (0.85, 0.96)		0.90 (0.83, 0.97)	
Health insurance status		0.7769		0.3497
Has	0.92 (0.88, 0.96)		0.93 (0.88, 0.98)	
Non	0.91 (0.85, 0.98)		0.89 (0.81, 0.97)	
Smoked at least 100 cigarettes		0.0118		0.4950
Yes	0.87 (0.81, 0.93)		0.90 (0.83, 0.98)	
No	0.97 (0.91, 1.02)		0.94 (0.87, 1.01)	

ORs were calculated as per 10 scores increase in LE8 score.

## 4 Discussion

Utilizing the NHANES database, we performed a cross-sectional investigation to explore the correlation between LE8 scores representing CVH and HPV infection. Our results suggest that individuals with elevated LE8 scores exhibited a reduced odds of HPV and HR-HPV infection. Subgroup analysis revealed that marital status influenced the relationship between CVH and HPV as well as HR-HPV infections, with married or cohabiting individuals experiencing greater benefits from higher CVH scores. Smoking history also influenced the relationship between CVH and HPV infection, with smokers benefiting more from higher LE8 scores. Furthermore, in the case of HR-HPV infection, individuals with tertiary education rarely benefit from higher LE8 scores.

To the best of our knowledge, this research represents the first attempt to investigate the association between CVH as quantified by LE8 score and HPV infection. The LE8 score encompasses eight health behaviors and factors impacting CVH, providing an intuitive assessment of peoples’ cardiovascular well-being. Previous researches had predominantly focused on exploring the correlation between CVD and HPV infection and suggested that HPV infection is positively associated with CVD (7, 8). In a large-scale study conducted in Korea, 63,411 Korean women without pre-existing CVD were enrolled. Researchers found a strong link between HR-HPV infection and a higher incidence of CVD in women. Those who tested positive had hazard ratios (HR) compared to those who tested negative (HR = 1.25). Furthermore, the presence of obesity was found to elevate the risk of developing CVD (HR = 1.73) (11). In a 17-year follow-up study, researchers found that women with HR-HPV infection had a higher mortality risk after developing CVD compared to those who were not infected. What is more, obesity reinforces this risk (19). However, a study on head and neck squamous cell carcinoma patients found that HPV-negative patients had a higher 5-year cumulative incidence of CVD compared to HPV-positive patients (20). In another study, HPV DNA was detected in the atherosclerotic coronary arteries of 20 deceased patients who had suffered from myocardial infarction, with 55% of the samples testing positive for HR-HPV (21). In our study, it was observed that a 10 points enhance in CVH score was correlated with a 8% decrease in the positivity rates of HPV and HR-HPV, after adjusting for all covariates. Furthermore, it was noted that BMI did not exert any influence on this inverse relationship, which was different from previous studies. We also investigated the relationship between BP and HPV infection, given that atherosclerosis directly affects BP. Our results indicated a negative correlation between BP and HR-HPV infection only in the unadjusted model; however, this correlation disappeared after adjusting for covariates. This negative correlation observed in the unadjusted model may be influenced by confounding factors. The relationship between CVH and HPV infection appears to be more complex, as BP could be associated with other underlying variables, such as age, race, BMI and smoking history.

The mechanism behind the negative correlation between CVH and HPV infection is complex and currently lacks consensus. It is mainly believed to be the result of chronic infection. HPV infection can induce systemic inflammation and lead to an increase in inflammatory mediators in circulation, thereby fostering the development of atherosclerosis (19, 22). In addition, HPV, as a virus that primarily infects epithelial cells, may also directly infect vascular endothelial cells (23). Some studies have demonstrated that HPV can potentially access arterial tissues via the systemic circulation (21, 24, 25). The expression of vascular endothelial growth factor was controlled by the HPV through its oncoprotein E6, thus participating in the process of atherosclerosis (26). Meanwhile, the HPV oncoproteins E6 and E7 can degrade the tumor suppressor protein P53, which accelerates atherosclerosis, as P53 plays a crucial role in regulating this process (22, 27).

Furthermore, HPV infection is correlated with several shared risk factors with CVD. As widely recognized, tobacco exposure poses a significant risk for CVD and is related to the occurrence of severe cardiovascular events (28). Concurrently, previous studies have shown that smoking impairs the body’s ability to eliminate the HPV virus. This leads to persistent infection and ultimately contributes to a poor prognosis for cervical cancer (29–31). In our study, participants who had a history of smoking, exhibit a stronger negative correlation between CVH and HPV infection. At the same time, the socioeconomic status of individuals also exerts an influence on the prevalence of both CVD and HPV infection (32, 33). We also found that, the level of education influenced the relationship between CVD and HR-HPV infection. There are several other common influences, including hyperlipidemia, obesity, physical activity, etc. (34–36). These findings suggested the potential effectiveness of employing the LE8 score as an indicator for assessing CVH and HPV infection. And the observed correlation between CVH and HPV infection may stem from common risk factors.

This study has several advantages. Firstly, the data employed in this research was obtained from the nationally conducted survey NHANES, thereby affording a substantial sample size and a degree of representativeness. Secondly, this study controlled for a series of covariates associated with HPV infection and CVH, enhancing the credibility of the fully adjusted model. Thirdly, we investigated the impact of various factors on the correlation between CVH and HPV infection through subgroup analysis. This study is subject to several limitations. First, the cross-sectional design of the study does not allow for the determination of a causal relationship between CVH and HPV infection. Whether CVH and HPV infection are causally related or their correlation stems from common risk factors needs to be exposed in future in-depth prospective studies. Second, while several covariates were incorporated into the analysis, the possibility remains that pertinent covariates were overlooked. Finally, owing to restrictions inherent in the NHANES database, the generalizability of the study’s findings may be constrained. Therefore, future comparative studies in other countries and regions are necessary.

## 5 Conclusion

In summary, the findings of this study illustrated a negative association between CVH and HPV infection, as well as HR-HPV infection. The LE8 score shows potential as a shared predictive biomarker for both CVH and HPV infection. More prospective studies are needed to delve deeper into the causal association between CVH and HPV infection, as well as the underlying mechanisms.

## Data Availability

The datasets presented in this study can be found in online repositories. The names of the repository/repositories and accession number(s) can be found at: https://www.cdc.gov/nchs/nhanes/.
