# Supplementary material for: Association between cardiovascular health and human papillomavirus infection: analysis from NHANES 2005–2016
Source: Front Public Health. 2024 Nov 22;12:1501409. doi: 10.3389/fpubh.2024.1501409 (PMC11621051; doi:10.3389/fpubh.2024.1501409)
Supplement: Supplementary file 1 [file Data_Sheet_1.docx]

**Supplementary Table S1. Healthy Eating Index-2015 Components**

| Component | Maximum points | Standard for maximum score | Standard for minimum score of zero |
| --- | --- | --- | --- |
| Adequacy | | | |
| Total Fruits[^2^](https://epi.grants.cancer.gov/hei/developing.html#f2) | 5 | ≥0.8 cup equivalents/1,000 kcal | No Fruit |
| Whole Fruits[^3^](https://epi.grants.cancer.gov/hei/developing.html#f3) | 5 | ≥0.4 cup equivalents/1,000 kcal | No Whole Fruit |
| Total Vegetables[^4^](https://epi.grants.cancer.gov/hei/developing.html#f4) | 5 | ≥1.1 cup equivalents/1,000 kcal | No Vegetables |
| Greens and Beans[^4^](https://epi.grants.cancer.gov/hei/developing.html#f4) | 5 | ≥0.2 cup equivalents/1,000 kcal | No Dark Green Vegetables or Legumes |
| Whole Grains | 10 | ≥1.5 oz equivalents/1,000 kcal | No Whole Grains |
| Dairy[^5^](https://epi.grants.cancer.gov/hei/developing.html#f5) | 10 | ≥1.3 cup equivalents/1,000 kcal | No Dairy |
| Total Protein Foods[^6^](https://epi.grants.cancer.gov/hei/developing.html#f6) | 5 | ≥2.5 oz equivalents/1,000 kcal | No Protein Foods |
| Seafood and Plant Proteins[^6^](https://epi.grants.cancer.gov/hei/developing.html#f6)^,^[^7^](https://epi.grants.cancer.gov/hei/developing.html#f7) | 5 | ≥0.8 oz equivalents/1,000 kcal | No Seafood or Plant Proteins |
| Fatty Acids[^8^](https://epi.grants.cancer.gov/hei/developing.html#f8) | 10 | (PUFAs + MUFAs)/SFAs ≥2.5 | (PUFAs + MUFAs)/SFAs ≤1.2 |
| Moderation | | | |
| Refined Grains | 10 | ≤1.8 oz equivalents/1,000 kcal | ≥4.3 oz equivalents/1,000 kcal |
| Sodium | 10 | ≤1.1 grams/1,000 kcal | ≥2.0 grams/1,000 kcal |
| Added Sugars | 10 | ≤6.5% of energy | ≥26% of energy |
| Saturated Fats | 10 | ≤8% of energy | ≥16% of energy |

PUFAs, polyunsaturated fatty acids; MUFAs, monounsaturated fatty acids; cSFAs, saturated fatty acids.

**Supplementary Table S2. Life’s Essential 8 Components and Criteria for Scoring**

| **Domain** | **CVH Metric** | **Measurement** | **Quantification and Scoring of CVH Metric** |
| --- | --- | --- | --- |
| Health Behaviors | Diet | Healthy Eating Index-2015 diet score  Example tools: 24-hour dietary recall | Quantiles of DASH-style diet adherence  Scoring (Population):  Points Quantile  100 ≥95^th^ percentile (top/ideal diet)  80 75^th^ – 94^th^ percentile  50 50^th^ – 74^th^ percentile  25 25^th^ – 49^th^ percentile  0 1^st^ – 24^th^ percentile (bottom/least ideal quartile) |
|  | Physical activity | Self-reported minutes of moderate or vigorous physical activity per week  Example tools: NHANES PAQ | Metric: Minutes of moderate (or greater) intensity activity per week  Scoring:  Points Minutes  100 ≥150  90 120 – 149  80 90 – 119  60 60 – 89  40 30 – 59  20 1 – 29  0 0 |
|  | Nicotine exposure | Self-reported use of cigarettes or inhaled nicotine- delivery system  Example tools: NHANES SMQ and SMQFAM | Metric: Combustible tobacco use and/or inhaled NDS use; or secondhand smoke exposure  Scoring:  Points Status  100 Never smoker  75 Former smoker, quit ≥5 yrs  50 Former smoker, quit 1 - <5 yrs  25 Former smoker, quit <1 year, or currently using inhaled NDS  0 Current smoker  Subtract 20 points (unless score is 0) for living with active indoor smoker in home |
|  | Sleep health | Self-reported average hours of sleep per night  Example tools: NHANES SLQ | Metric: Average hours of sleep per night  Scoring:  Points Level  100 7 – <9  90 9 – <10  70 6 – <7  40 5 – <6 or ≥10  20 4 – <5  0 <4 |
| Health Factors | Body mass index | Body weight (kg) divided by height squared (m^2^)  Example tools: NHANES BMX | Metric: Body mass index (kg/m^2^)  Scoring:  Points Level  100 <25  70 25.0 – 29.9  30 30.0 – 34.9  15 35.0 – 39.9  0 ≥40.0 |
|  | Blood lipids | Plasma total and HDL-cholesterol with calculation of non-HDL-cholesterol  Example tools: NHANES TCHOL, HDL and BPQ | Metric: Non-HDL-cholesterol (mg/dL)  Scoring:  Points Level  100 <130  60 130 – 159  40 160 – 189  20 190 – 219  0 ≥220  If drug-treated level, subtract 20 points |
|  | Blood glucose | Fasting blood glucose or casual hemoglobin A1c  Example tools: NHANES GHB and DIQ | Metric: Fasting blood glucose (mg/dL) or Hemoglobin A1c (%)  Scoring:  Points Level  100 No history of diabetes and FBG <100 (or HbA1c < 5.7)  60 No diabetes and FBG 100 – 125 (or HbA1c 5.7-6.4) (Pre-diabetes)  40 Diabetes with HbA1c <7.0  30 Diabetes with HbA1c 7.0 – 7.9  20 Diabetes with HbA1c 8.0 – 8.9  10 Diabetes with Hb A1c 9.0 – 9.9  0 Diabetes with HbA1c ≥10.0 |
|  | Blood pressure | Appropriately measured systolic and diastolic blood pressure  Example tools: NHANES BPX and BPQ | Metric: Systolic and diastolic blood pressure (mm Hg)  Scoring:  Points Level  100 <120/<80 (Optimal)  75 120-129/<80 (Elevated)  50 130-139 or 80-89 (Stage I HTN)  25 140-159 or 90-99  0 ≥160 or ≥100  Subtract 20 points if treated level |

Abbreviations: HDL, high-density lipoprotein; NDS, nicotine-delivery system; NHANES, National Health and Nutrition Examination Surveys.

**Supplementary Table S3 The association between BP and HPV infection**

|  | **Model 1**  **OR (95% CI) *P*-value** | **Model 2**  **OR (95% CI) P-value** | **Model 3**  **OR (95% CI) P-value** |
| --- | --- | --- | --- |
| **HPV infection status** |  |  |  |
| systolic blood pressure | 1.00 (1.00, 1.00) 0.8439 | 1.00 (1.00, 1.00) 0.2783 | 1.00 (1.00, 1.00) 0.4017 |
| Tertile 1 | Reference | Reference | Reference |
| Tertile 2 | 0.98 (0.87, 1.09) 0.6640 | 0.97 (0.87, 1.09) 0.6153 | 0.97 (0.87, 1.09) 0.6421 |
| Tertile 3 | 0.98 (0.88, 1.09) 0.7230 | 1.04 (0.92, 1.17) 0.5384 | 1.03 (0.91, 1.17) 0.6278 |
| *P* for trend | 0.7507 | 0.4902 | 0.5842 |
| Diastolic blood pressure | 1.00 (0.99, 1.00) 0.2559 | 1.00 (1.00, 1.00) 0.6961 | 1.00 (1.00, 1.01) 0.5403 |
| Tertile 1 | Reference | Reference | Reference |
| Tertile 2 | 0.98 (0.88, 1.09) 0.7126 | 1.05 (0.94, 1.18) 0.4099 | 1.06 (0.94, 1.19) 0.3592 |
| Tertile 3 | 0.93 (0.84, 1.04) 0.2045 | 1.02 (0.92, 1.15) 0.6652 | 1.04 (0.93, 1.16) 0.5186 |
| *P* for trend | 0.1976 | 0.6944 | 0.5423 |
| **HR-HPV infection status** |  |  |  |
| systolic blood pressure | 0.99 (0.99, 1.00) 0.0012 | 1.00 (0.99, 1.00) 0.5241 | 1.00 (0.99, 1.00) 0.5161 |
| Tertile 1 | Reference | Reference | Reference |
| Tertile 2 | 0.83 (0.72, 0.95) 0.0082 | 0.85 (0.74, 0.98) 0.0262 | 0.85 (0.74, 0.99) 0.0322 |
| Tertile 3 | 0.83 (0.72, 0.95) 0.0075 | 0.99 (0.85, 1.15) 0.9089 | 1.00 (0.85, 1.17) 0.9693 |
| *P* for trend | 0.0120 | 0.9995 | 0.9503 |
| Diastolic blood pressure | 0.99 (0.99, 1.00) 0.0068 | 1.00 (0.99, 1.00) 0.7830 | 1.00 (0.99, 1.01) 0.9111 |
| Tertile 1 | Reference | Reference | Reference |
| Tertile 2 | 0.93 (0.81, 1.08) 0.3451 | 1.02 (0.89, 1.19) 0.7436 | 1.03 (0.89, 1.19) 0.6957 |
| Tertile 3 | 0.81 (0.71, 0.93) 0.0033 | 0.96 (0.83, 1.11) 0.6173 | 0.97 (0.84, 1.13) 0.7256 |
| *P* for trend | 0.0030 | 0.6050 | 0.7146 |

Model 1 took no adjustments;

Model 2 adjusted for age and race;

Model 3 adjusted for age, race, BMI and smoking history.
